# Mapping Error Propagation in Intraoral Scanning Using Reason’s Swiss-Cheese Model: An In Vitro Study of Precision Under Repeatability Conditions

**DOI:** 10.3390/dj14050267

**Published:** 2026-05-04

**Authors:** Cristina-Alexandra Cozmescu, Ana Maria Cristina Țâncu, Lucian Toma Ciocan, Vlad Gabriel Vasilescu, Ana Cernega, Silviu-Mirel Pițuru, Marina Imre

**Affiliations:** 1 Discipline of Prosthodontics, Faculty of Dentistry, “Carol Davila” University of Medicine and Pharmacy, 37 Dionisie Lupu Street, District 2, 020021 Bucharest, Romania; cristina-alexandra.balan@drd.umfcd.ro (C.-A.C.); anamaria.tancu@umfcd.ro (A.M.C.Ț.); marina.imre@umfcd.ro (M.I.); 2 Discipline of Dental Prosthesis Technology, Faculty of Dentistry, “Carol Davila” University of Medicine and Pharmacy, Dionisie Lupu Street, No. 37, District 2, 020021 Bucharest, Romania; 3 Department of Organization, Professional Legislation and Management of the Dental Office, Faculty of Dental Medicine, “Carol Davila” University of Medicine and Pharmacy, 17–23 Plevnei Street, 020021 Bucharest, Romania; ana.cernega@umfcd.ro (A.C.); silviu.pituru@umfcd.ro (S.-M.P.)

**Keywords:** intraoral scanner, digital impressions, precision, repeatability, root mean square, Swiss-cheese model, Reason, error mitigation, quality control

## Abstract

**Background:** Intraoral scanning (IOS) errors seldom originate from a single-point failure; instead, they arise from interactions among hardware performance, reconstruction software, and operator-dependent acquisition behaviors. James Reason’s layered defense (Swiss-cheese) model provides a systems-oriented lens to trace how residual vulnerabilities can propagate into clinically relevant surface distortions. **Objectives:** To quantify within-scanner precision of IOS under repeatability conditions in a controlled in vitro setting and to propose a Reason-based framework that maps defensive layers, barriers, and residual failure modes along the IOS workflow. **Methods:** A controlled in vitro design was implemented to minimize clinical confounders. A standardized partially edentulous maxillary reference specimen was scanned repeatedly with three IOS systems under fixed environmental conditions using a standardized scanning strategy. Within each IOS, precision was quantified from repeated scans using surface deviation metrics, including root mean square (RMS) deviation, percentile-based dispersion, and the percentage of points within a predefined tolerance band. Residual vulnerabilities were organized into a systems-oriented error framework by defensive layer (hardware, software/processing, and acquisition/operator) and workflow stage (pre-scan preparation, acquisition, reconstruction/registration, and export/verification). **Results:** Deviation-based precision metrics revealed scanner-specific dispersion patterns, including differences in RMS magnitude and tail behavior (percentile spread), suggesting scanner-specific patterns of residual distortion under the tested conditions. Tolerance-based metrics further showed that threshold selection materially influences interpretability and perceived clinical relevance. In vitro IOS precision assessed under repeatability conditions should be interpreted as an emergent output of multiple interacting defensive layers rather than as the isolated performance of a single component. Coupling deviation-based precision metrics with Reason’s layered defense model yields a clinically actionable framework for quality control, helping anticipate where residual risk is most likely to accumulate and where mitigation checkpoints can be implemented. **Conclusions:** In vitro IOS repeatability should be interpreted as an emergent output of multiple interacting defensive layers rather than the isolated performance of a single component. Coupling repeatability metrics with Reason’s layered defense model supports a framework for quality-oriented interpretation, helping anticipate where residual risk is most likely to accumulate and where mitigation checkpoints can be implemented.

## 1. Introduction

The digital transformation of dentistry has been propelled by a persistent goal: to improve procedural standardization and reduce variability attributable to human factors, environmental conditions, and external dependencies. Because many restorative and implant outcomes are sensitive to fine morphology and micrometric discrepancies, controlling avoidable error sources is particularly relevant. Nonetheless, the complete elimination of inaccuracies across clinical workflows remains unlikely, as deviations can emerge from complex interactions among operator-related behaviors, device performance, reconstruction algorithms, and intraoral conditions. Within this context, characterizing the probability, mechanisms, and spatial distribution of acquisition-related error is essential for a multidisciplinary evaluation of digital scanning processes.

Digital workflows in dentistry emerged from the development of CAD/CAM concepts, which enabled the transition from conventional impressions to virtual models. Chairside digital dentistry expanded with the introduction of early clinical systems and, later, with the rapid diversification of intraoral scanner (IOS) platforms and their repeated hardware and software updates. Over the last decade, optical acquisition principles such as triangulation, confocal imaging, and structured light projection have matured, while clinical applications have expanded from single-unit restorations to orthodontics, implant dentistry, and diagnostic documentation. Accordingly, contemporary evaluations of IOS performance should consider not only the optical acquisition principle, but also the reconstruction pipeline and software versioning [1,2,3,4,5].

The clinical adoption of IOS systems in prosthodontics has increased because they can streamline workflows, improve patient comfort, and integrate directly with CAD/CAM manufacturing. However, their clinical utility depends on the accuracy of three-dimensional surface capture, particularly in implant-supported rehabilitations, where small deviations may translate into clinically relevant misfit. IOS accuracy is influenced not only by device design, but also by scanning strategy, operator experience, calibration status, and intraoral environmental conditions. Current evidence indicates that these outcomes cannot be inferred from manufacturer specifications alone, which supports the need for standardized evaluations under controlled conditions [6,7,8,9].

According to ISO 5725-1:2023, “ISO 5725 uses two terms ‘trueness’ and ‘precision’ to describe the accuracy of a measurement method” [10]. In the present study, only precision under repeatability conditions was assessed, using repeated scan-to-scan surface comparisons.

A key challenge is that intraoral scans may accumulate both systematic and random distortions as scan span increases and landmark continuity decreases, making it difficult to separate intrinsic hardware performance from software-based stitching/registration behavior and procedural effects. In implant workflows, additional complexity arises from scan-body geometry and the fidelity of the corresponding CAD library, both of which may influence deviation patterns. Taken together, these observations suggest that scan quality is better understood as the output of a multistep workflow rather than as a fixed attribute of a scanner model [11,12,13,14,15,16].

**Figure 1:**
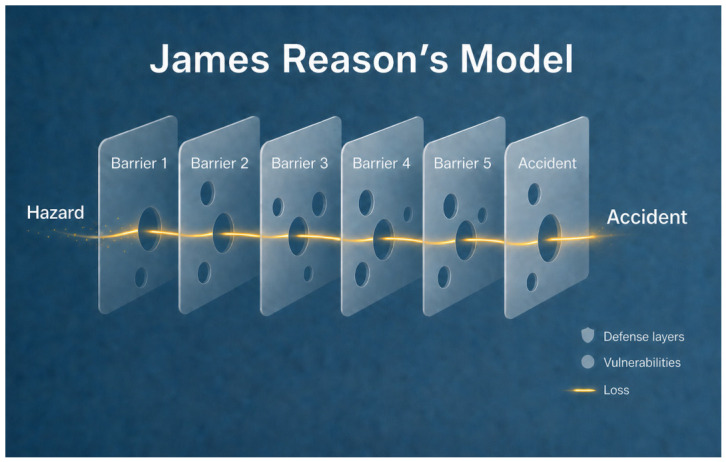
Swiss-cheese model of accident causation (Reason). Conceptual schematic included to support visualization of the theoretical framework applied in the present study. The initial draft was generated using OpenAI ChatGPT Images 2.0, accessed through ChatGPT (OpenAI, San Francisco, CA, USA), and was subsequently and scientifically refined by the authors for academic use. Defense layers are represented by the translucent barriers, vulnerabilities by the circular openings, and loss by the yellow trajectory passing through aligned vulnerabilities. The figure is illustrative only and does not represent primary study data [17].

James Reason (1938–2025) proposed a layered view of error in complex systems, distinguishing active failures from latent conditions that shape the probability of adverse outcomes. In his ‘Swiss-cheese’ model, undesirable events do not usually result from a single failure, but from the alignment of vulnerabilities distributed across multiple defensive layers. In healthcare and medical-device contexts, this perspective is consistent with contemporary risk-management and usability principles, which emphasize interactions among technology, users, tasks, and environment rather than hardware specifications alone [17,18]. A conceptual representation of Reason’s Swiss-cheese model is shown in Figure 1.

Within patient-safety science and human factors engineering, the Swiss-cheese model remains influential because it provides a practical framework for distinguishing frontline failures from latent organizational or design-related weaknesses [19,20,21,22]. Evidence from healthcare safety research further supports that adverse events commonly arise from multi-step breakdowns distributed across complex workflows rather than from isolated single-point failures [23,24,25]. This systems-oriented perspective is transferable to digital dentistry, where IOS deviations may similarly emerge from interacting technical, procedural, software-related, and operator-dependent vulnerabilities.

Overall, IOS accuracy is shaped by both device-related and context-dependent variables, including scanner selection, operator performance, calibration, anatomical complexity, and environmental constraints. As scan span increases, particularly in partially edentulous arches, cumulative stitching errors may become more pronounced and may be difficult to disentangle from software processing effects. In implant dentistry, scan-body geometry, CAD-library fidelity, software version, and scanning strategy may further contribute to variability. These considerations underline the importance of detailed methodological reporting and support the use of structured error classifications that distinguish operator-related from patient-related or system-related components [6,26,27].

Extending the scan span in implant-supported or partially edentulous arches increases the likelihood of cumulative registration drift, particularly when morphological landmarks are discontinuous and inter-implant distances are large. Evidence syntheses indicate that trueness tends to deteriorate as scan length increases, whereas the influence of moderators such as scanning strategy, operator experience, and scan-body design remains heterogeneous across studies. Accordingly, the present study focuses on within-scanner precision under repeatability conditions as a pragmatic way to characterize the stability of the scanner–software pipeline, while acknowledging that full-arch trueness benchmarking was outside the present scope [28].

### Research Questions

The study addressed the following research questions: (i) Do repeated scans acquired with the same IOS system under controlled conditions show distinct repeatability profiles as quantified by RMS- and percentile-based dispersion metrics? (ii) Can repeatability distributions be discussed as scanner-specific “risk signatures” that reflect residual vulnerabilities across defensive layers (hardware, software/processing, and acquisition workflow) as conceptualized by Reason’s Swiss-cheese model? (iii) Which workflow checkpoints appear most relevant as potential mitigation barriers, based on the observed patterns of dispersion and tail behavior?

## 2. Materials and Methods

### 2.1. Study Design

**Figure 2:**
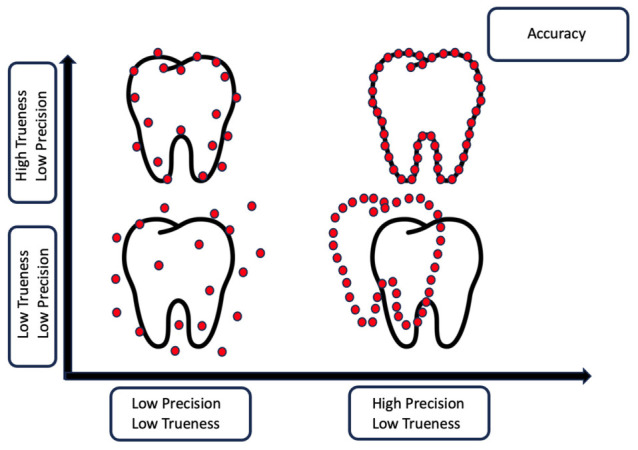
Conceptual distinction between trueness and precision in digital impressions. Schematic created by the authors based on ISO definitions [10]. The black tooth outline represents the true reference geometry, whereas the red points represent repeated measurement or scanning points. High trueness indicates closeness of the measured points to the reference geometry, while high precision indicates clustering of repeated measurements. The figure is schematic and was created by the authors for conceptual illustration; it does not represent experimental scan data from the present study.

This controlled in vitro study was designed (i) to evaluate within-scanner precision of intraoral scanning under repeatability conditions and (ii) to interpret these precision outcomes using a system-oriented framework grounded in Reason’s layered defense (Swiss-cheese model). The in vitro setting was intentionally selected to minimize clinical confounders (e.g., saliva, soft-tissue mobility, involuntary patient movements, and gag reflex), thereby isolating variability attributable to the scanning chain itself (device optics/sensors, reconstruction and registration algorithms, and acquisition workflow). In accordance with ISO terminology, the study assessed precision under repeatability conditions. Whole-arch trueness assessment was outside the scope of the present protocol because it requires a traceable, independently validated metrological reference for the entire specimen, consistent with ISO definitions of accuracy components [10]. The conceptual distinction between trueness and precision is illustrated in Figure 2.

### 2.2. Reference Specimen and Scan-Body Geometry

**Figure 3:**
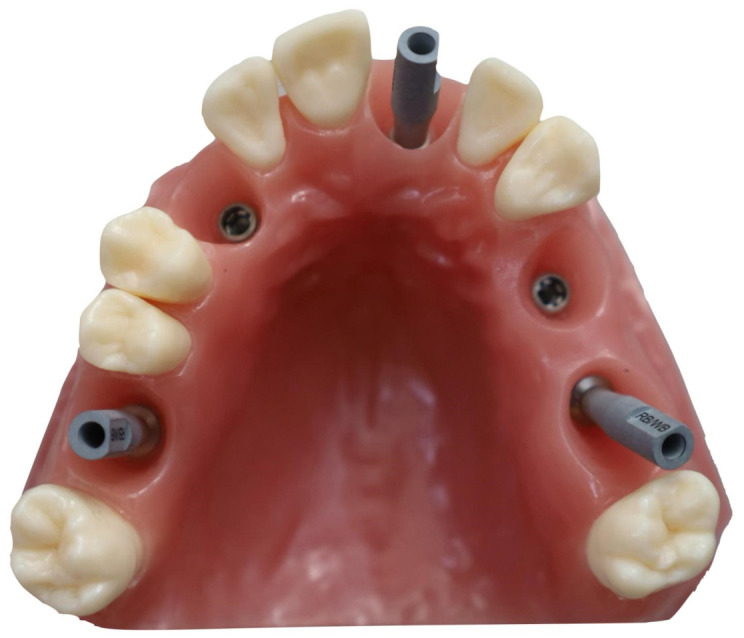
Partially edentulous maxillary model with five titanium implants positioned at 1.6, 1.3, 2.1, 2.4, and 2.5. The scan-body/scan-component geometries positioned at 1.6, 2.1, and 2.5 were used for alignment and analysis.

A dimensionally stable partially edentulous maxillary model was used as the reference specimen. The specimen geometry, including prepared tooth surfaces and implant scan bodies, was selected to reflect clinically relevant features that are susceptible to stitching drift and local registration errors. The configuration of the reference specimen, including the distribution of implant positions and the scan-body positions, is shown in Figure 3. All scans were acquired under standardized environmental conditions and using a predefined acquisition strategy to reduce operator-induced variability. Scanning was performed by a single trained operator. Scanner calibration and/or manufacturer-recommended verification procedures were applied consistently throughout the acquisition period, and device settings were maintained according to manufacturer guidance [26,29,30].

### 2.3. Intraoral Scanners (IOS)

Three IOS systems were evaluated to represent commonly used platforms while incorporating distinct optical acquisition architectures: TRIOS 5 (3Shape, Copenhagen, Denmark), Medit i700 (Medit Corp., Seoul, Republic of Korea), and Planmeca Emerald S (Planmeca, Helsinki, Finland). TRIOS 5 employs confocal imaging principles with ScanAssist guidance and is available in a wireless configuration. Medit i700 is based on structured-light acquisition and supports open export formats (e.g., STL/PLY/OBJ); an i700 Wireless version is marketed separately. Planmeca Emerald S utilizes projected-pattern triangulation with multi-wavelength illumination and a heated tip intended to reduce fogging.

These scanners were selected to support comparative interpretation across different optical engines and workflow ecosystems, without implying a market-share-driven selection. Prior studies have reported clinically acceptable performance for these platforms in full-arch and implant-related scanning scenarios, although outcomes vary by study design, scan span, and evaluation method [31,32,33,34,35].

To provide a structured technical overview, hardware characteristics are summarized in Table 1, including acquisition principle, illumination/capture descriptors, device mass, connectivity, anti-fog and asepsis-related features, and field of view (FOV). Specifications were compiled from manufacturer/distributor sources and are reported for contextual comparison rather than analytical weighting.

### 2.4. Scanning Strategy

A standardized full-arch scanning protocol was applied consistently across all acquisitions. The scan path followed a closed-loop, three-pass strategy consisting of sequential occlusal, buccal (vestibular), and palatal passes along the entire arch. The protocol included capture of the posterior palatal vault and the vibrating line (AH line) to support global loop closure.

During the occlusal pass (1.7 → 2.6), the scanner tip was maintained at an approximate working distance of 10–20 mm from the surface. The operator performed slow, continuous motion with deliberate overlap between successive frames to preserve stable tracking. When scan bodies were encountered, specifically at positions 1.6, 2.1, and 2.5, a brief circumferential 360° acquisition was performed using a short helical motion around each scan body to capture multiple facets/planes and to strengthen local registration [36].

Following the occlusal lane, the buccal (vestibular) pass was completed in reverse direction (2.6 → 1.7). This approach was selected to increase overlap and to create acquisition loops across hemi-arches, to help limit long-range drift that may occur when continuous acquisition extends over larger distances without loop closure. Evidence indicates that scanning strategy, particularly segmentation, overlap, and loop-closure behavior, can influence accuracy outcomes in extended-arch acquisitions [37,38,39,40].

The protocol concluded with the palatal pass (1.7 → 2.6). Palatal surfaces were captured with consistent overlap; local re-scanning was performed only when software feedback indicated incomplete coverage or discontinuities. The extended palate and AH line were then captured to reinforce global alignment and facilitate loop closure. Prior investigations suggest that inclusion of palatal reference areas may improve alignment stability in full-arch acquisitions, although the effect may depend on palatal anatomy and vault morphology [41].

### 2.5. Environmental Standardization and Reproducibility

All acquisitions were performed under standardized laboratory conditions by a single operator to minimize inter-operator variability. Before each acquisition block, manufacturer recommendations were followed to ensure stable performance. For systems requiring routine calibration, calibration was performed as specified by the manufacturer; for systems marketed as calibration-free, manufacturer-recommended verification and readiness checks were performed [42].

Software versions were kept constant throughout the experiment to avoid confounding from mid-study algorithmic updates [43]. Ambient illumination was maintained within a typical clinical operatory range while avoiding extremes known to destabilize optical tracking [42,44,45,46,47,48,49].

The study was conducted in a climate-controlled environment maintained at 23 ± 1 °C and 45–55% relative humidity, monitored continuously with a calibrated thermohygrometer. The reference model was acclimatized to the same environment for at least 30 min prior to scanning to reduce potential optical artifacts related to temperature gradients or condensation. Environmental control was implemented because relative humidity and ambient temperature changes have been associated with measurable effects on IOS accuracy and acquisition behavior in prior reports [50,51].

### 2.6. Software Workflow and Deviation Analysis

All scan datasets were analyzed using Medit Design within the Medit Link v3.4 software environment (Medit Corp., Seoul, Republic of Korea). Surface-to-surface comparisons were performed using the Compare/Deviation Display module to enable alignment, deviation mapping, and extraction of quantitative deviation indicators. Separate project groups were created for each scanner (TRIOS 5, Medit i700, Planmeca Emerald S), and datasets were imported via Medit Link to ensure systematic file traceability. To minimize software-handling bias across devices, all datasets were exported in the same open mesh format (STL) and imported into Medit Link using manufacturer default export settings. Prior to deviation analysis, meshes underwent minimal, standardized cleaning/trimming to remove non-anatomical artifacts (isolated islands, spikes, and peripheral over-extensions), without smoothing, decimation, hole filling, or re-meshing; identical alignment and deviation settings were then applied to all scanner groups.

Within each scanner-specific group, one scan was pre-specified as the reference model using an a priori rule to reduce subjective selection bias. The reference scan was defined as the first dataset meeting predefined completeness criteria (continuous coverage of areas of interest, absence of gross acquisition interruptions, and no need for manual mesh repair). The remaining 19 scans were compared exclusively within the same group (intra-group comparisons only). Thus, each scanner generated 19 intra-group overlays, and scanner groups were analyzed separately throughout, without pooling datasets across devices. The “Align target data separately” setting was enabled to avoid progressive error accumulation during repeated alignment operations.

**Figure 4:**
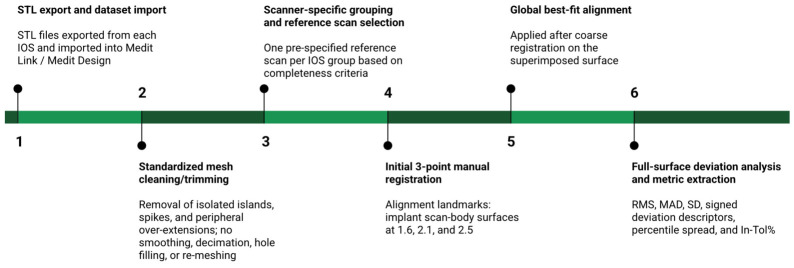
Schematic overview of the scan-analysis workflow used for intra-group overlay comparisons. STL datasets were exported and imported into Medit Design, minimally cleaned using a standardized protocol, grouped by scanner, and compared against one pre-specified reference scan per IOS group. Coarse registration was initialized by three-point manual alignment at scan-body positions 1.6, 2.1, and 2.5, followed by global best-fit alignment and full-surface deviation analysis. Quantitative outputs included RMS, MAD, SD, signed deviation descriptors, percentile-based spread, and In-Tol%.

Alignment followed a coarse-to-fine registration strategy. In the first stage, a three-point manual registration was performed using the implant scan-body surfaces at positions 1.6, 2.1, and 2.5 (FDI notation) as alignment reference landmarks. These geometrically stable, non-collinear positions were selected to define a triangulated spatial configuration spanning both quadrants and the anterior region, thereby constraining all six degrees of freedom during initial pose estimation and providing a reliable starting position for the subsequent iterative optimization. In the second stage, a global best-fit alignment was applied across the entire superimposed surface. Best-fit alignment corresponds conceptually to iterative minimization of point-to-surface deviations (ICP-like optimization) as commonly applied in 3D metrology and digital dentistry. The alignment reference landmarks were used exclusively for initialization of the coarse registration step; after subsequent global best-fit alignment, deviation visualization and quantitative extraction were performed on the full superimposed surface. Representative full-surface deviation maps generated under identical display settings are presented in Section 3 as qualitative complements to the overlay-level numerical summaries. The overall scan-analysis workflow used for intra-group overlay comparisons is summarized schematically in Figure 4.

For each overlay, quantitative deviation indicators were extracted from the point-to-surface deviation field to characterize within-group precision under repeatability conditions. RMS deviation was used as a global descriptor of deviation magnitude; mean absolute deviation (MAD) was recorded as a complementary summary less sensitive to outliers; and the standard deviation (SD) of the deviation distribution captured dispersion around the mean. In addition, an in-band percentage (In-Tol%) was computed as the proportion of surface points with an absolute deviation ≤ 0.050 mm (≤50 μm), providing a threshold-based indicator of the density of deviations within a predefined tolerance band. This metric panel was selected to capture both magnitude- and distribution-related aspects of precision using surface-overlay methodologies commonly reported in IOS precision studies.

Medit Design/Compare was selected as the analytic platform because it has been frequently employed as a validated tool for surface-based alignment and deviation quantification in in vitro IOS research. The literature supports robustness of results when best-fit alignment is applied consistently and when deviation indicators such as RMS, MAD, and percent in-band are systematically reported. Moreover, several studies have explicitly referenced Medit Design v3.1.x as a primary software environment for overlay comparisons, supporting its appropriateness as a standardized analytical tool for the present evaluation [52,53].

### 2.7. Statistical Analysis

Deviation indicators (RMS, MAD, SD, and In-Tol%) were extracted for each overlay within each scanner-specific group and summarized across repeated scans to characterize within-scanner precision under repeatability conditions. Continuous outcomes were reported using descriptive statistics (mean and standard deviation, and, where appropriate, median and interquartile range) to reflect distributional behavior. No formal hypothesis testing was planned. This choice was consistent with the exploratory scope of the study, which constitutes an initial attempt to operationalize Reason’s layered defense model for the systematic interpretation of IOS precision variability. Because the primary objective was to establish and propose a systems-oriented interpretative architecture rather than to confirm between-device hierarchies, a descriptive analytical framework was considered the most appropriate starting point. Additionally, within each scanner group, all overlay-level deviation summaries share a single pre-specified reference scan, introducing a shared-anchor dependency that may not fully satisfy the independence assumptions of standard between-group tests; the use of different reference scans across groups further means that between-group differences could partly reflect anchor-related effects. Comparisons among IOS systems were therefore interpreted descriptively, with emphasis on relative magnitude, dispersion behavior, and tolerance-based profiles rather than on inferential between-group ranking. In-Tol% was calculated using the predefined absolute deviation threshold of ≤0.050 mm (≤50 μm).

### 2.8. Error Classification and Quality-Control Framework

To standardize interpretation of IOS outcomes beyond numerical deviation metrics, the workflow incorporated an a priori error-classification framework intended to capture predictable sources of distortion during acquisition and processing and to support subsequent Reason-based interpretation. Errors were organized into four domains: (A) hardware selection-related errors, including scanner-dependent reliability variability and implant scan-body (ISB) geometry alterations associated with component selection or mechanical change; (B) software and processing-related errors, including scan noise and algorithm-related over-contouring/altered ISB morphology that may affect mesh-to-library correspondence; (C) patient-related errors, operationalized as humidity-related disturbances and general scanning artifacts known to impair optical stability and surface recognition; and (D) operator-related errors, subdivided into pre-scan preparation failures (e.g., “bridge-type” artifacts, unclear/diffuse finishing lines, and soft-tissue interposition) and acquisition-stage errors (e.g., scanning-process artifacts, mesh holes, stitching/merging artifacts, distance-related errors, and scan-strategy-induced mesh deformation). The structure and subdomains of this a priori classification framework are presented in Table 2. This framework was applied as a pre-export quality-control checklist to guide targeted re-scanning and dataset acceptance/rejection, and to provide a reproducible vulnerability map for linking deviation patterns to system-layer weaknesses within the Swiss-cheese model during the Discussion. The subsequent use of this framework in the Discussion was intended as an interpretive and hypothesis-generating aid grounded in the literature, rather than as empirically validated one-to-one attribution of specific deviation metrics to discrete error layers within the present dataset [14,27,37,54,55,56,57].

### 2.9. Use of Generative AI in Figure Preparation

During the preparation of this manuscript, the authors used OpenAI ChatGPT Images 2.0, accessed through ChatGPT (OpenAI, San Francisco, CA, USA) solely to create an initial conceptual draft for Figure 1. The figure was included only as an illustrative aid to support visualization of Reason’s Swiss-cheese model as the theoretical framework underpinning the present study; it does not represent primary study data, analytical output, or empirical results. The generated draft was subsequently critically reviewed, substantially edited, and scientifically refined by the authors, who take full responsibility for the final content of the figure and of the manuscript. No generative AI tool was used for data acquisition, deviation analysis, statistical processing, interpretation of the results, or formulation of the study conclusions.

## 3. Results

To support standardized interpretation of the quantitative outputs used in this study, Table 3 summarizes the principal deviation indices applied in the analysis. For each parameter, the table provides a definition and practical interpretation in deviation assessment, together with a hypothesis-driven mapping to error domains that could plausibly influence each metric (interpretive aid, not causal attribution).

Within-scanner precision outcomes obtained under repeatability conditions are summarized in Table 4. One pre-specified reference scan per scanner group was independently overlaid with each of the remaining 19 scans, yielding 19 overlays for Medit i700, 19 for TRIOS 5, and 19 for Emerald S. Table 4 reports overlay-level summaries of RMS, MAD, SD, percentile-based spread, signed deviation descriptors, and In-Tol%, thereby characterizing the precision profile of each scanner under the standardized workflow and predefined alignment reference regions.

**Figure 5:**
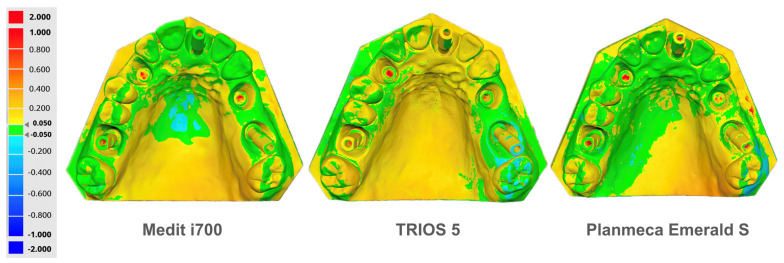
Representative full-surface color deviation maps from intra-group overlay comparisons for the three IOS systems (Medit i700, TRIOS 5, Planmeca Emerald S), displayed under identical deviation settings. Green indicates deviations within ±0.050 mm, whereas warmer and cooler colors indicate positive and negative deviations, respectively. The ±2.0 mm extremes correspond to the software display range and do not imply identical true extreme deviations across scanners.

Representative full-surface color deviation maps from intra-group overlay comparisons are shown in Figure 5 under identical display settings for the three IOS systems. Although intended as qualitative visual complements rather than quantitative endpoints, these maps appear consistent with the dispersion profiles reported in Table 4.

Across the scanned pattern, Medit i700 showed the lowest overall dispersion (RMS 0.088 mm; 88 μm), followed by Emerald S (RMS 0.119 mm; 119 μm) and TRIOS 5 (RMS 0.139 mm; 139 μm). The corresponding absolute average deviations were 0.049 mm (49 μm), 0.063 mm (63 μm), and 0.073 mm (73 μm), respectively. Percentile-based spread, expressed as (90–10)/2, showed the same rank order (0.075 mm; 75 μm for Medit i700; 0.088 mm; 88 μm for Emerald S; and 0.101 mm; 101 μm for TRIOS 5). The tolerance-based In-Tol% also differed across devices (64.78% for Medit i700; 57.02% for Emerald S; and 52.83% for TRIOS 5) when applying the predefined absolute deviation band of ≤0.050 mm.

The exported distribution descriptors suggested a mild negative shift in central tendency across all groups (negative signed averages), consistent with a slight asymmetry in the deviation distributions. Specifically, the 10th percentile was farther from zero than the 90th percentile for all scanners (Medit i700: −81 μm vs. +68 μm; Emerald S: −104 μm vs. +72 μm; TRIOS 5: −132 μm vs. +71 μm).

Minimum and maximum values were reported at approximately −2.0 mm and +2.0 mm for all groups, reflecting the software reporting/display limit applied to the deviation range in the global deviation output rather than indicating identical extreme deviations across scanners. The repeatability magnitudes observed were comparable to the 3D deviation ranges reported in previous clinical full-arch implant impression studies (e.g., 139 ± 56 μm to 185 ± 81 μm, depending on implant number), supporting the plausibility of the dispersion levels seen in this controlled workflow [58,59].

## 4. Discussion

The purpose of this Discussion is to move interpretation beyond a strictly metrological reading of the precision outputs obtained under repeatability conditions and to situate the findings within a complex socio-technical workflow. Rather than treating each outcome as an intrinsic property of a scanner, we use James Reason’s layered defense (“Swiss-cheese”) model as a conceptual and systems-oriented interpretive framework through which residual variability in intraoral scanning may be discussed. Within this perspective, clinically meaningful deviation may be considered as potentially arising from the interaction of multiple weaknesses distributed across several defensive layers, although establishing such interactions empirically would require designs beyond the scope of the present study.

### 4.1. Hardware Selection-Related Errors

**Objective (defensive intent).**

Within the Swiss-cheese model, hardware selection acts as an upstream defensive layer intended to provide a stable acquisition platform and constrain variability before it propagates through stitching, post-processing, or operator-dependent decisions. In implant prosthodontics, this intent extends to the implant scan-body (ISB) system, which serves as geometric reference for implant position transfer and CAD library alignment [60].

**Barriers (implemented safeguards).**

Safeguards at this layer typically include evidence-guided scanner selection according to clinical indication (e.g., short-span vs. extended-span/full-arch scans), systematic calibration/maintenance routines, verification of hardware integrity (scanner tip condition and optical cleanliness), and software/firmware version control to reduce untracked performance drift. In parallel, ISB-related safeguards include use of manufacturer-validated scan bodies, consistent seating and tightening, and selection of ISB geometries that reduce ambiguity during reconstruction and library matching. In complete-arch implant scans, stabilization strategies such as scan-body splinting or artificial landmarks have been shown to influence accuracy outcomes [61].

**Vulnerabilities (residual failure modes within the layer).**

Despite formal safeguards, hardware-level defenses may retain latent weaknesses that become measurable as reduced precision across repeated scans, specifically scanner-dependent precision variability and ISB-related deviations that could compromise mesh-to-library congruence. Within the layered-defense logic, such residual failure modes may increase the likelihood that later layers are less able to compensate.

**Interpretation of surface deviation metrics in relation to the present findings.**

Because this study quantified within-scanner precision under repeatability conditions, hardware-layer vulnerabilities may be most plausibly reflected in dispersion and tolerance-sensitive indices (RMS, SD, Abs Avg, (90–10)/2, and In-Tol%). In this dataset, the scanner group with the most constrained dispersion also showed higher tolerance compliance (Medit i700: RMS 0.088 mm; Abs Avg 0.049 mm; (90–10)/2 0.075 mm; In-Tol 64.78%), whereas greater dispersion corresponded to lower in-band percentage (TRIOS 5: RMS 0.139 mm; (90–10)/2 0.101 mm; In-Tol 52.83%; Emerald S: RMS 0.119 mm; (90–10)/2 0.088 mm; In-Tol 57.02%). Under standardized acquisition and analysis conditions, these patterns appear compatible with device-specific precision characteristics, suggesting that hardware selection may function as a foundational barrier contributing to the precision baseline observed under the present conditions.

**Literature context.**

Evidence syntheses consistently emphasize that IOS outcomes depend on device-related factors and that performance cannot be inferred from nominal manufacturer specifications alone. Furthermore, the importance of controlling device readiness and calibration status is recurrent in clinical guidance and experimental designs that seek to isolate scanner–software behavior. Together, these findings support a Reason-informed interpretation: hardware selection does not eliminate error but can reduce residual variability, strengthening the likelihood that downstream layers will intercept deviations before they accumulate into clinically relevant distortion [6,14,31].

### 4.2. Software- and Processing-Related Errors

**Objective (defensive intent).**

Within Reason’s model, the software/processing layer may be interpreted as responsible for transforming optical measurements into a geometrically faithful surface mesh, preserving self-consistency across tracking, stitching/registration, triangulation, filtering and scan-body recognition. In implant workflows, scan-body regions may serve as geometric constraints; therefore, even subtle algorithmic deviations affecting scan-body morphology could exert disproportionate downstream effects during CAD library alignment and implant position transfer.

**Barriers (implemented safeguards).**

At this layer, safeguards are embedded within proprietary reconstruction pipelines and may include tracking stabilization and loop closure, coverage monitoring, noise filtering, management of topological consistency, and use of validated scan-body libraries. In the present study, additional “barriers” were introduced by standardization at the analysis stage: scanner-specific group analyses (no cross-device overlays), best-fit alignment under the same workflow, consistent alignment-region constraints, and consistent metric reporting (RMS/MAD/SD, percentiles, In-Tol%). These steps reduce the risk that observed differences arise from heterogeneous post-processing choices.

**Vulnerabilities (residual failure modes within the layer).**

Software-driven failure modes may remain clinically relevant because they can present as subtle distribution shifts rather than obvious defects. Within the proposed framework, these vulnerabilities may be associated primarily with scan noise/instability and algorithm- or library-mediated alteration of scan-body morphology (e.g., smoothing/rounding of facets, local “bulging,” or altered contours). Conceptually, these phenomena may plausibly influence deviation distributions differently: noise would tend to broaden dispersion, whereas systematic contour alteration could increase directional asymmetry and tail behavior [60].

**Interpretation of surface deviation metrics in relation to the present findings.**

Because comparisons were performed within scanner groups, the deviation indices describe how consistently each scanner–software pipeline reproduces the same geometry across repeats. Noise-related instability may be interpreted as being reflected in spread- and outlier-sensitive metrics (SD, RMS, Abs Avg, (90–10)/2 and In-Tol%). The dispersion rank order observed in the Results, together with the mild negative shift in central tendency across groups, should be interpreted as descriptive signatures of repeatability behavior under the selected workflow conditions rather than as evidence for a specific underlying mechanism.

**Literature context.**

The literature supports that IOS “accuracy” is partly software-governed and therefore not a purely hardware-constant property. A controlled investigation reported that software updates can significantly affect both trueness and precision, with effects that may be positive or negative depending on the device and version [40].

Related work demonstrates that algorithmically mediated procedures during acquisition and reconstruction can reshape outcomes: rescanning mesh holes and stitching procedures have been reported to decrease trueness and precision, with the number and dimensions of rescanned areas influencing accuracy [35,36,45,48].

Collectively, these findings can be discussed as consistent with the layered-defense logic: software and processing may function as a stabilizing barrier but may also amplify residual vulnerabilities, especially when corrective actions (e.g., rescanning/patching) introduce new registration trajectories rather than purely repairing missing data [35,45,48].

### 4.3. Patient-Related Errors

**Objective (defensive intent).**

The patient-related layer may be discussed as a clinically unavoidable contextual stratum whose defensive function is to preserve a stable optical environment so that acquisition and registration remain consistent. In restorative and implant prosthodontics, moisture dynamics and soft-tissue behavior may impair scan continuity and compromise scan-body capture even when device and software performance are otherwise robust.

**Barriers (implemented safeguards).**

Safeguards at this layer are primarily procedural: isolation, surface drying, control of fogging, management of bleeding when applicable, soft-tissue retraction, and patient positioning to reduce access limitations and motion. In the present study, patient-layer threats were intentionally suppressed by the in vitro design, creating a “best-case” baseline for scanner repeatability under controlled conditions.

**Vulnerabilities (residual failure modes within the layer).**

In vivo, patient-related vulnerabilities persist as residual failure modes that can degrade scanning consistency, particularly through moisture/humidity-related disturbances (fluid films, saliva pooling, fogging) and scanning artifacts related to access constraints and soft-tissue interference.

**Interpretation relative to the present findings.**

Because this dataset was generated in vitro, patient-related vulnerabilities were not expected to dominate repeatability metrics. Nevertheless, the metric structure used here offers a conceptually coherent basis for clinical projection: activating patient-layered variability could plausibly broaden dispersion (SD, (90–10)/2), increase outlier-sensitive metrics (RMS), and reduce In-Tol% by increasing the density of points outside the tolerance band, although these expectations would require empirical confirmation under in vivo conditions.

**Literature context (clinical relevance).**

Evidence supports the clinical impact of wet conditions. A 2024 study reported that the presence of saliva significantly affects the accuracy of digital implant transfer, with scan-body characteristics interacting with wettability conditions [62].

A meta-analysis of in vitro studies similarly found that digital impression accuracy improved under dry conditions, and also reported effects of scan pattern/strategy [38,44].

Complementary implant-focused experimental data also indicate that humidity and drying conditions can influence trueness and precision in complete-arch implant scans [50,55].

Together, these findings may support discussing patient-related conditions as an active layer in the Swiss-cheese interpretation rather than as incidental nuisance variables [12,38,55,63].

### 4.4. Operator-Related Errors

**Objective (defensive intent).**

Within Reason’s Swiss-cheese framework, the operator layer may be interpreted as responsible for preserving acquisition stability and procedural consistency, maintaining tracking integrity, adequate overlap, stable working distance, and anatomically faithful surface capture. This intent is particularly stringent in implant workflows, where small acquisition deviations may be amplified through registration and mesh reconstruction.

**Barriers (implemented safeguards).**

Barriers at this layer include (i) a standardized scanning strategy, (ii) disciplined control of scanning speed, angulation, and scanner-to-surface distance, (iii) deliberate overlap management to preserve tracking continuity, and (iv) structured quality-control behaviors, such as real-time verification of coverage and selective rescanning restricted to essential regions. Evidence further indicates that clinician- and workflow-dependent variability can meaningfully affect digital outcomes, supporting the role of protocol-driven safeguards and training as operator-level defenses. In the present study, operator-layer barriers were reinforced through experimental standardization (single trained operator; fixed scan path; controlled environment; fixed software versions) and by post-acquisition verification using distribution-based surface deviation metrics and tolerance compliance indices [64,65].

**Vulnerabilities (residual failure modes within the layer).**

Despite safeguards, operator-related vulnerabilities remain clinically meaningful because they may occur intermittently, present as localized distortions, or accumulate into global registration drift. In the proposed a priori framework, these residual failure modes can be organized along the workflow timeline:

- Pre-scan preparation vulnerabilities (subtypes 4.1–4.4) including general pre-scan preparation-related vulnerabilities such as bridge-type artifacts, unclear or diffuse preparation margins and soft-tissue interposition, which may adversely affect acquisition boundary conditions, particularly under clinical constraints [27,66].
- Acquisition-stage execution vulnerabilities (subtypes 4.5, 4.8, 4.10) reflecting inconsistency in movement smoothness, overlap discipline and distance control [67,68].
- Reconstruction-sensitive procedural vulnerabilities (subtypes 4.6, 4.7), particularly mesh holes and stitching/merging errors arising from corrective actions [15,69,70].
- Cumulative drift patterns (subtype 4.9) including long-span “umbrella-type” distortions that are difficult to detect visually but emerge in quantitative mapping [27,71].
- Strategy-dependent deformation mechanisms (subtype 4.11) driven by scan path selection and landmark progression over extended spans [29,30,47].

Taken together, these subtypes represent a continuum of operator-driven vulnerabilities that may remain contained by robust defenses or, under certain conditions, align with hardware- and software-layer limitations and thereby reduce workflow resilience [37,72].

**Interpretation of surface deviation metrics in relation to the present findings (operator layer).**

Given that the present study quantified within-scanner repeatability (precision) through repeated scan-to-scan overlays, the deviation metrics can be interpreted as quantitative signatures of how effectively the operator layer preserved acquisition stability under controlled conditions, and how these signatures would be expected to evolve in more variable clinical environments.

- Dispersion-sensitive indicators such as SD and (90–10)/2 may be particularly responsive to operator-driven inconsistency (4.5 scanning-process variability, 4.10 distance instability, 4.8 operator-dependent reliability, and 4.11 strategy heterogeneity). As these vulnerabilities intensify, deviation distributions broaden even when the signed mean remains near zero.
- Outlier-weighted indicators such as RMS (and extreme values when uncapped) may be particularly sensitive to episodic destabilization and defect amplification, including (4.6) mesh holes and (4.7) stitching/merging inconsistencies, and may increase disproportionately when localized regions become severely distorted. The emergence of (4.9) umbrella-type drift could contribute to higher RMS values beyond what is suggested by robust spread metrics alone.
- Directionality descriptors (Avg(+), Avg(−), 90th/10th percentiles) capture whether operator-related vulnerabilities create asymmetry in deviation distributions. For example, (4.2) bridge-type artifacts and (4.4) soft-tissue interposition might tend to elevate positive excursions, whereas systematic under-capture, contraction bias, or strategy-driven drift (4.11) may preferentially reinforce the negative tail.
- Tolerance compliance (In-Tol%) may function as an operational proxy for defensive performance and may decrease as operator-related vulnerabilities become more frequent or spatially extensive across the surface.

In the present in vitro dataset, acquisition was standardized and performed by a single trained operator, which likely limited the contribution of technique variability relative to device–software repeatability signatures. Nonetheless, the metric structure used here may offer a transferable basis to clinical interpretation: as clinical complexity activates pre-scan preparation (4.1–4.4) and increases execution variability (4.5–4.11), a conceptually coherent expectation, to be confirmed by future in vivo investigations, would be broader dispersion (higher SD and interdecile spread), increased outlier sensitivity (higher RMS) and reduced tolerance compliance (lower In-Tol%).

**Literature context.**

The operator contribution to IOS outcomes is widely recognized: inter-operator variability can influence both trueness and precision, particularly over extended scan spans and in implant workflows. These findings may be discussed as consistent with the operator layer acting as a defense whose effectiveness depends on protocol adherence and training [37,38,67,68,70,72,73,74].

**Limitations of the Study**

Several limitations should be acknowledged. First, this investigation was deliberately scoped to within-scanner precision under repeatability conditions in a controlled in vitro setting. Consequently, the reported outcomes should be interpreted as precision profiles derived from surface overlay deviations, rather than as absolute accuracy rankings. Whole-arch trueness assessment was outside the scope of the protocol because it would require an independent, traceable, and validated metrological reference for the entire specimen and trueness estimates may also be influenced by the selection of the reference dataset and the test object [73].

Second, repeatability estimates were generated using a single-reference scan approach within each scanner-specific group. Although the reference scan was defined using an a priori completeness rule to reduce subjective selection bias, single-anchor designs may remain partially dependent on the chosen reference dataset and therefore cannot fully exclude anchor-related effects.

Third, deviation analysis relied on surface-based best-fit registration (with alignment-region constraints when applied). Best-fit alignment may redistribute deviations and attenuate certain global discrepancy components; accordingly, the overlays should be interpreted as descriptors of repeatability behavior under standardized alignment conditions rather than as metrological trueness estimates. Although mesh cleaning/trimming was standardized and applied equally across groups, any manual post-processing may introduce operator-dependent variability and can influence deviation metrics by altering the analyzed surface area. Future studies could compare raw versus cleaned datasets or use automated, protocol-driven trimming to further reduce this source of variability. To preserve internal comparability, identical alignment settings and decision rules were applied across scanners [75].

Fourth, the study design intentionally minimized clinical confounders (e.g., saliva, soft-tissue dynamics, and patient movement) and applied a standardized acquisition protocol performed by a single trained operator. While this strengthens internal validity and isolates scanner–software repeatability behavior, it limits direct extrapolation to intraoral conditions in which patient- and operator-layer vulnerabilities may be more prominent and may interact with reconstruction algorithms.

Fifth, deviation quantification was performed in a single software environment (Medit Design/Compare); although the same workflow and parameters were applied to all devices, software-specific import/processing behavior may influence deviation estimates, and cross-validation with an independent metrology platform could strengthen generalizability. Prior to deviation analysis, meshes were minimally trimmed/cleaned using a predefined protocol to remove non-anatomical artifacts (loose islands, spikes, and peripheral over-extensions) while preserving the clinically relevant surfaces and the scan-body regions. The same software tools, operator, and criteria were used for all scanners; no smoothing, hole filling, decimation, or re-meshing was performed. Deviation metrics were computed on the cleaned/trimmed meshes.

Sixth, the tolerance-based indicator In-Tol% (≤0.050 mm/50 μm) is inherently threshold-dependent. It was therefore interpreted as complementary to continuous dispersion descriptors (RMS/MAD/SD and percentile-based spread), rather than as a universal clinical acceptability criterion.

Notwithstanding the above, the limitations of the present study should be interpreted in the context of its exploratory scope. As the first investigation, to the best of the authors’ knowledge, to operationalize Reason’s layered defense model for intraoral scanning, the work was designed to establish a conceptual foundation rather than to provide confirmatory evidence. This is consistent with the broader maturation of Reason’s framework across safety-critical domains, where conceptual mapping preceded quantitative operationalization. The identified constraints therefore simultaneously define the directions along which future research may extend and strengthen the proposed framework.

## 5. Conclusions

**Figure 6:**
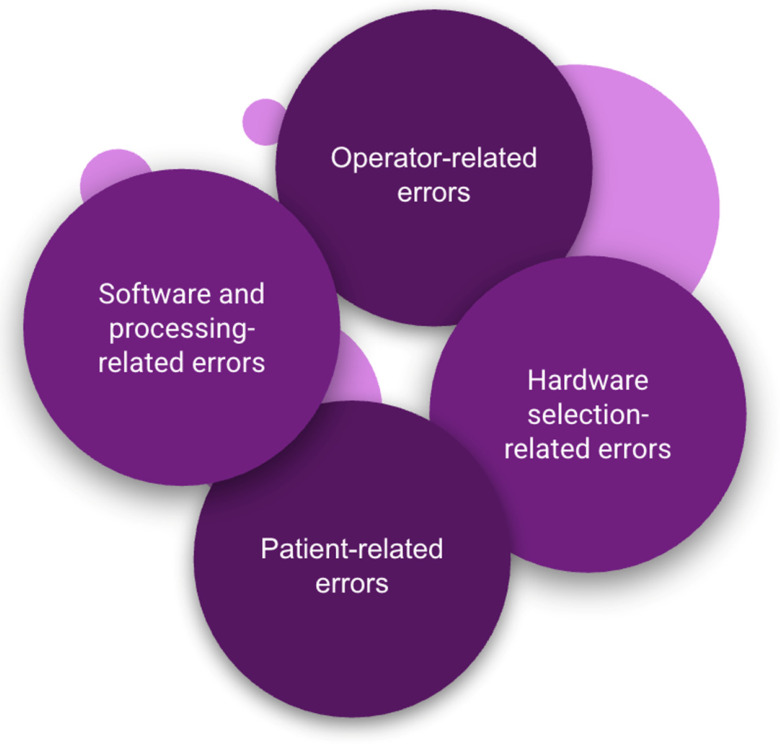
Diagram illustrating the proposed Swiss-cheese-inspired error framework for intraoral scanning, organized into four interacting domains: hardware selection, software/processing, patient-related, and operator-related factors. The overlap between layers highlights how scan inaccuracies may emerge from alignment of residual vulnerabilities across domains rather than from a single isolated cause.

This in vitro investigation supports the applicability of Reason’s layered defense (Swiss-cheese) model to intraoral scanning by showing that scan quality is best interpreted as an emergent output of multiple interacting defensive layers rather than as the isolated performance of a single component. By structuring the workflow into hardware selection, software/processing, patient-related, and operator-related strata, the proposed error-classification framework provides a systems-oriented lens for interpreting precision outcomes obtained under repeatability conditions and for anticipating where residual risk is most likely to accumulate during digital impression acquisition in prosthodontic and implant-supported workflows. The proposed Swiss-cheese-inspired framework for intraoral scanning is summarized in Figure 6.

From a risk-management perspective, the present results reinforce that clinically relevant deviations are unlikely to be explained by one dominant failure mode. Instead, deviations become consequential when residual vulnerabilities align across layers, for example, operator-dependent strategy and distance variability interacting with reconstruction-sensitive behaviors (mesh discontinuities and stitching/merging instability) and implant scan-body recognition constraints. Accordingly, the principal contribution of this study is the establishment of a Reason-informed quality-control framework that translates deviation distributions into interpretable “risk signatures”, enabling proactive mitigation through defense-in-depth interventions (standardized scan strategy, disciplined distance control, restrained and rule-based rescanning, calibration governance, validated scan-body selection, and objective tolerance-based monitoring alongside continuous dispersion descriptors).

Collectively, these findings support the clinical utility of integrating surface deviation statistics into routine digital workflow governance, not as a tool to rank devices, but as a practical method to detect early degradation of defensive performance and to strengthen error containment before deviations propagate into prosthetic design decisions and implant position transfer. Future research should extend this framework to in vivo environments and incorporate an external reference model to jointly quantify trueness and precision, thereby refining the relationship between layered vulnerabilities, repeatability signatures, and clinically meaningful restorative outcomes [76].

## Figures and Tables

**Table 1:** Technical characteristics of the IOS systems included in the study.

	TRIOS 5 (3Shape)	Medit i700	Planmeca Emerald S
Acquisition principle	Confocal imaging with ScanAssist intelligent alignment	Structured-light/triangulation	Projected-pattern triangulation
Capture Rate and Illumination	LED; up to ~2400 images/s	LED; up to ~70 FPS	RGN lasers; >67 3D datasets/s (video capture)
Weight	~299 g (including battery)	~245 g (handpiece)	229 g (scanner with tip); 339 g with cable
Connectivity and Wireless options	Wireless connectivity (TRIOS 5 Wireless)	Available as a separate i700 Wireless model; i700 base is wired	No (USB-A/C cable)
Anti-fog and infection control	Closed hygienic tip design; autoclavable/ready-tip options	Active anti-fog; UV-C LED; reversible tip; autoclavable	Actively heated tip; autoclavable tips
Field of view (FOV)	Not specified by manufacturer	Tip scanning area ~15 × 13 mm	17.6 × 13.2 mm
Operating temperature (°C)	15–26	18–28	15–28
Operating humidity % Relative Humidity (RH)	10–85	20–75	5–95

**Table 2:** A priori error-classification framework for intraoral scanning in implant prosthodontics, organized by system layer (hardware, software, patient, and operator domains).

1. Hardware Selection-Related Errors	2. Software- and Processing-Related Errors	3. Patient-Related Errors	4. Operator-Related Errors
1.1. Reliability errors (depending on scanner selection)	2.1. Scan noise	3.1. Humidity-related errors	4.1. Pre-scan preparation-related errors
1.2. ISB geometry deformations (depending on scan body selection)	2.2. Over-contouring and altered ISB morphology	3.2. Scanning artifacts	4.2. “Bridge-type” errors
			4.3. Unclear/diffuse preparation margins
			4.4. Soft-tissue interposition errors
			4.5. Scanning process-related errors
			4.6. Mesh holes
			4.7. Stitching/merging errors
			4.8. Reliability errors
			4.9. “Umbrella-type” errors
			4.10. Distance-related errors
			4.11. Scan-strategy-induced deformations

**Table 3:** Deviation indices used in the analysis: definitions and hypothesis-driven links to potential error domains.

Deviation Metric	Statistical Meaning	Interpretation in Surface Deviation Analysis	Error Domains Most Likely to Influence the Metric (Rationale)
Min.	Most negative deviation	Extreme negative outliers; may be affected by reporting limits	scanning artifacts; mesh holes; stitching/merging artifacts; distance/strategy-induced distortions; margin ambiguity
Max.	Most positive deviation	Extreme positive outliers; may be affected by reporting limits	over-contouring/altered ISB morphology; soft-tissue/interposition; bridging artifacts; stitching/merging artifacts; scan noise
Median	Midpoint of distribution	Central tendency; robust to extremes	systematic drift/registration bias; strategy-induced deformation; distance-related distortion
Avg.	Signed arithmetic mean	Global signed bias (contraction vs. expansion)	systematic stitching drift; over-contouring; ISB geometry/library mismatch; distance/strategy effects
Abs Avg.	Mean of absolute deviations	Global mismatch magnitude (sign-independent)	widespread noise/instability; cumulative stitching drift; humidity-related disturbances; workflow inconsistency
RMS	Root mean square	Global magnitude emphasizing larger deviations/outliers	severe local defects; stitching/merging artifacts; mesh discontinuities; tracking loss
Std. Dev.	Dispersion around mean	Width of deviation distribution	regional instability and heterogeneous error patterns; scan noise; humidity-related disturbances
Var.	(Std. Dev.)^2^	Derived dispersion metric	same interpretive domain as SD
Avg. (+)	Mean of positive deviations	Outward/overbuild tendency	over-contouring; bridging/tissue capture; overlap-induced positive bumps
Avg. (−)	Mean of negative deviations	Inward/under-capture tendency	under-capture; mesh holes; contraction drift; distance/strategy distortions
(90–10)/2	Half interdecile range	Robust spread (less sensitive to extremes)	broadly distributed inconsistency across the scan (vs. isolated outliers)
10th percentile	Lower-tail quantile	More negative with systematic inward bias over substantial areas	widespread drift/under-capture; strategy-induced distortion
90th percentile	Upper-tail quantile	More positive with systematic outward bias over substantial areas	overbuild/bridging; tissue capture; widespread overlap
In-Tol%	Proportion within tolerance	Threshold-based stability indicator; depends on tolerance	any factor increasing deviation density; sensitive to generalized instability and drift

Note: The listed error domains represent a literature-informed interpretive mapping designed to support the proposed framework; they do not constitute causal attribution derived from the present in vitro dataset.

**Table 4:** Overlay-level repeatability metrics for the 19 intra-group comparisons performed within each IOS group.

Properties	Medit i700	TRIOS 5	Emerald S
Min.	−2.000 mm	−2.000 mm	−2.000 mm
Max.	1.986 mm	1.998 mm	1.996 mm
Median	−0.004 mm	−0.012 mm	−0.009 mm
Avg.	−0.008 mm	−0.029 mm	−0.017 mm
Abs Avg.	0.049 mm	0.073 mm	0.063 mm
RMS	0.088 mm	0.139 mm	0.119 mm
Std. Dev.	0.088 mm	0.136 mm	0.118 mm
Var.	0.008 mm^2^	0.019 mm^2^	0.014 mm^2^
Avg. (+)	0.045 mm	0.051 mm	0.053 mm
Avg. (−)	−0.053 mm	−0.089 mm	−0.071 mm
(90–10)/2	0.075 mm	0.101 mm	0.088 mm
10th percentile	−0.081 mm	−0.132 mm	−0.104 mm
90th percentile	0.068 mm	0.071 mm	0.072 mm
In-Tol%	64.78%	52.83%	57.02%

## Data Availability

The datasets generated and analyzed during the current study, including overlay-level deviation outputs and the associated processed mesh files used for comparative analysis, are available from the corresponding authors upon reasonable request. Data are not deposited in a public repository at this stage because of file-size and workflow-format constraints.

## Abbreviations

The following abbreviations are used in this manuscript:

AH line	Vibrating line (“AH line”)
AI	Artificial Intelligence
CAD/CAM	Computer-Aided Design/Computer-Aided Manufacturing
FOV	Field of View
FPS	Frames per second
ICP	Iterative Closest Point
In-Tol%	In-tolerance percentage
IOS	Intraoral scanner(s)
ISB	Implant Scan Body
ISO	International Organization for Standardization
ISO/TR 20896-2	ISO Technical Report 20896-2
LED	Light-Emitting Diode
MAD	Mean Absolute Deviation
OBJ	Wavefront OBJ
PLY	Polygon File Format
RH	Relative Humidity
RMS	Root Mean Square
SD	Standard Deviation
STL	Standard Tessellation Language
USB-A/C	USB Type-A/Type-C
UV-C	Ultraviolet C
